# A telomere-to-telomere genome assembly for *Malassezia pachydermatis* ATCC 14522 (CBS 1879)

**DOI:** 10.1128/mra.00125-25

**Published:** 2025-06-24

**Authors:** Lois L. Hoyer, Clarissa P. Souza, Chien-Che Hung, Elizabeth K. Hogan, Alvaro G. Hernandez

**Affiliations:** 1 Department of Pathobiology, College of Veterinary Medicine, University of Illinois Urbana-Champaign 14589, Urbana, Illinois, USA; 2 Department of Veterinary Clinical Medicine, College of Veterinary Medicine, University of Illinois Urbana-Champaign 14589, Urbana, Illinois, USA; 3 Veterinary Diagnostic Laboratory, University of Illinois Urbana-Champaign 14589, Urbana, Illinois, USA; 4 Roy J. Carver Biotechnology Center, University of Illinois Urbana-Champaign 14589, Urbana, Illinois, USA; University of Maryland School of Medicine, Baltimore, Maryland, USA

**Keywords:** otitis externa, yeast, canine, genome assembly

## Abstract

Pacific Biosciences (PacBio) long-read sequencing was used to generate a telomere-to-telomere genome assembly for the *Malassezia pachydermatis* type strain ATCC 14522 (CBS 1879). The assembly included six chromosomes. The nuclear genome was 8.4 Mb, with a GC content of 55.3%, and 4,519 predicted genes.

## ANNOUNCEMENT

*Malassezia* species (Kingdom Fungi, phylum Basidiomycota, subphylum Ustilaginomycotina, class Malasseziomycetes) are lipophilic yeasts associated with skin and mucosal sites of warm-blooded vertebrates (1, 2). *Malassezia pachydermatis* is commonly identified in canine skin and ear infections (2) and infrequently causes systemic disease in humans (3). The type strain ATCC 14522 (CBS 1879) was collected from a canine otitis externa case in Sweden. Although two genome sequences for the *M. pachydermatis* type strain are available in GenBank (4), they are highly fragmented. The goal of this work was to use Pacific Biosciences (PacBio) long-read sequencing technology to derive a telomere-to-telomere assembly for the *M. pachydermatis* type strain.

ATCC 14522 was purchased from the American Type Culture Collection (Manassas, VA, USA) and grown to saturation at 35°C in modified Dixon medium (mDixon; ATCC Medium 2693). The cells were collected by centrifugation, washed in sterile water, and resuspended in 3 mL 10 mM Tris-HCl pH 8.0 containing 100 mM NaCl, 10 mM MgCl_2_, and 2 mM 2-mercaptoethanol. An equal volume of phenol/chloroform (1:1) and a 0.5 mL equivalent of sterile glass beads (425–600 µm) were added to the tube. The tube was vortexed at top speed for 2 min and then centrifuged at 3,000 × *g* to separate the phases. The aqueous phase was collected, and DNA was precipitated with two volumes of isopropanol. DNA was resuspended in 10 mM Tris-EDTA (pH 8.0) and then treated with RNase A (Gold Biotechnology) at 37°C for 1 h. Proteinase K (Gold Biotechnology) was added and incubated at 50°C for 16 h. The preparation was extracted with phenol/chloroform, and DNA was precipitated with two volumes of isopropanol.

Genomic DNA was sheared to an average size of 13 kb using a Megaruptor 3 (Diagenode). Sheared DNA was run on a Blue Pippin system (Sage Science) to select 7–50 kb fragments using a 0.75% agarose cassette and DNA marker S1. Sheared fragments were converted to a library using the SMRTbell prep kit 3.0 (Pacific Biosciences). The library was sequenced on a single-molecule real-time (SMRT) cell on a PacBio Revio system using the circular consensus sequencing (CCS) mode and a 30 h movie time. CCS analysis used SMRT Link v13.1 (ccs --min-passes 3 --min-rq 0.99). The data set included 778,713 reads (10,935 bp mean read length).

Filtlong v0.1.2 (5) was used to select sequence reads greater than 10 kb that represented 671× coverage of the expected 8 Mb genome (4). The genome assembly was produced using hifiasm v0.19.6 with default parameters (6). Primary contigs, representing a collapsed haploid assembly, were used for further analysis. Hifiasm generated five gapless contigs, bounded by telomeric repeats (5′-TGTTAG-3′) that matched the predicted chromosome sizes for the *M. pachydermatis* karyotype (7, 8). The smallest chromosome was broken in the region that contained the rDNA repeated units. Two contigs, each with telomeric repeats at one end and rDNA repeated units at the other, were joined manually to generate the 0.77 Mb chromosome bounded by telomeric repeats, encoding five complete copies of the rDNA repeat. The completeness of the genome assembly was evaluated using BUSCO v5.3.2 (9). The percent complete and single-copy BUSCOs were 93% (fungi_odb10) and 89% (basidiomycota_odb10).

Transcriptome sequencing (RNA-seq) data were collected to aid gene model prediction. *M. pachydermatis* ATCC 14522 (CBS 1879) was grown on Emmons Modification Sabouraud Dextrose Agar (ATCC Medium 28) for 3 days at 35°C. Ten individual colonies were picked and used to inoculate 10 mL of either Emmons Modification Sabouraud Dextrose or modified Dixon medium in a 50 mL Erlenmeyer flask. The flasks were incubated at 30°C and 130 rpm shaking for 48 h. Ten milliliters of fresh growth medium were placed into a new 50 mL flask and inoculated with 1 mL from the starter culture. The flasks were incubated at 35°C and 200 rpm shaking for 3 h. Cells were collected by centrifugation, flash frozen in dry ice/ethanol, and stored at −80°C until RNA was extracted.

RNA was extracted using a hot phenol method (10). Genomic DNA was removed by DNase I treatment (Ambion), and RNA was purified using a QIAgen RNeasy kit. Paired-end RNA-seq libraries were prepared using the KAPA Hyper Stranded mRNA Library Kit (Roche). The libraries were pooled and quantitated by PCR. The libraries were sequenced on one SP lane for 251 cycles from each end of the cDNA fragments on a NovaSeq 6000 instrument (Illumina). Each sample produced approximately 13 million stranded read-pairs. The bcl2fastq v2.20 conversion software (Illumina) was used to generate and demultiplex fastq files and trim adapters from the 3’ end of each read.

The funannotate v1.8.17 package (11) was used to clean, sort, and mask the hifiasm nuclear genome assembly using default parameters. The funannotate train function was aided by the two RNA-seq data sets described above, and then gene models were created using funannotate predict. Predicted protein sequences were analyzed with InterProScan v5.67.88.0 (12) and eggNOG-mapper web v2.1.12 (13, 14). Both output files were passed to funannotate annotate.

The new genome assembly (GCA_047368035.1) predicted 4,519 genes, which is approximately 300 more than the current reference sequence (GCF_001278385.1) (Table 1). The new genome assembly provides a telomere-to-telomere, chromosome-level sequence that will advance research into *M. pachydermatis*-host interactions.

**TABLE 1:** Comparison of statistics for three *M. pachydermatis* ATCC 14522 (CBS 1879) genome assemblies

Feature	GCF_001278385.1	GCA_001264975.1	GCA_047368035.1
Total sequence length (bp)	8,150,648	8,158,346	8,403,632
Total ungapped length (bp)	8,149,729	8,157,565	8,403,632
No. of contigs	118	80	6
Contig N_50_ (bp)	642,545	643,831	1,455,848
No. of scaffolds	91	61	6
Size of the largest scaffold (bp)	1,489,072	1,423,774	1,836,604
Scaffold N_50_ (bp)	1,303,022	957,230	1,455,848
GC content (%)	55.0	55.0	55.5
Sequencing coverage (x)	250 x	463 x	671x^*a*^
No. of genes	4,202	–^*b*^	4519

^*a*^ PacBio sequence reads provided 1013x coverage of the *M. pachydermatis* genome. The GCA_047368035.1 assembly was created using filtlong v0.1.2 (5) to select reads with a minimum length of 10 kb that represented 671x genome coverage.

^*b*^ “–”, Genes were not annotated in this assembly.

## Data Availability

This whole-genome sequencing project was deposited in GenBank (PRJNA1186209). The genome assembly (ASM4736803v1) was given accession number GCA_047368035.1. Accession numbers CM104464.1 to CM104469.1 were assigned to the chromosomes described here. PacBio sequence reads were deposited in the Sequence Read Archive (SRR32256939). RNA-Seq reads were also deposited in the Sequence Read Archive (SRR31360621, SRR31360622). The project was assigned the locus prefix tag ACI68E (SAMN44750262).
